# Mycobacterium tuberculosis Utilizes Host Histamine Receptor H1 to Modulate Reactive Oxygen Species Production and Phagosome Maturation via the p38MAPK-NOX2 Axis

**DOI:** 10.1128/mbio.02004-22

**Published:** 2022-08-24

**Authors:** Siwei Mo, Jiubiao Guo, Taosheng Ye, Ximeng Zhang, Jiang Zeng, Yuzhong Xu, Bin Peng, Youchao Dai, Wei Xiao, Peize Zhang, Guofang Deng, Dechang Xu, Xiaoru Long, Yi Cai, Xinchun Chen

**Affiliations:** a Guangdong Provincial Key Laboratory of Regional Immunity and Diseases, Department of Pathogen Biology, School of Medicine, Shenzhen University, Shenzhen, China; b College of Pharmacy, Shenzhen Technology University, Shenzhen, China; c Pulmonary Diseases Department Two, National Clinical Research Center for Infectious Disease (Shenzhen), Guangdong Provincial Clinical Research Center for Infectious Diseases (Tuberculosis), Shenzhen Third People's Hospital, Southern University of Science and Technology, Shenzhen, China; d The Fifth People's Hospital of Ganzhou, Ganzhou, Jiangxi Province, China; e Department of Clinical Laboratory, Shenzhen Baoan Hospital, Shenzhen University, Shenzhen, China; f Department of Respiratory Medicine, Children’s Hospital of Chongqing Medical University, Chongqing Key Laboratory of Pediatrics, Chongqing, China; g National Clinical Research Center for Child Health and Disorders, Ministry of Education Key Laboratory of Child Development and Disorders, China International Science and Technology Cooperation Base of Child Development and Critical Disorders, Chongqing Key Laboratory of Pediatrics, Children’s Hospital of Chongqing Medical University, Chongqing, China; Max Planck Institute for Infection Biology

**Keywords:** *Mycobacterium tuberculosis*, HRH1, NOX2, p38MAPK, ROS

## Abstract

Tuberculosis (TB), which is caused by the single pathogenic bacterium, Mycobacterium tuberculosis, is among the top 10 lethal diseases worldwide. This situation has been exacerbated by the increasing number of cases of multidrug-resistant TB (MDR-TB) and extensively drug-resistant TB (XDR-TB). Histamine is an organic nitrogenous compound that mediates a plethora of cell processes via different receptors. The expression of histamine receptor H1 (HRH1), one of the four histamine receptors identified to date was previously reported to be augmented by M. tuberculosis infection, although the underlying mechanism is unclear. In the present study, we applied confocal microscopy, flow cytometry, and Western blotting to show that HRH1 expression was enhanced in macrophages following mycobacterial infection. Furthermore, by combining techniques of gene knockdown, immunoprecipitation, intracellular bacterial burden analysis, fluorescence labeling, and imaging, we found that M. tuberculosis targeted the host HRH1 to suppress NOX2-mediated cROS production and inhibit phagosome maturation and acidification via the GRK2-p38MAPK signaling pathway. Our findings clarified the underlying mechanism of the M. tuberculosis and host HRH1 interaction and may provide useful information for the development of novel antituberculosis treatments.

## INTRODUCTION

Tuberculosis (TB), which is caused by the pathogenic bacterium Mycobacterium tuberculosis, exerts a detrimental impact on public health, with latent infection occurring in approximately one-quarter of the global population (1). In 2020, TB was responsible for about 1.51 million deaths worldwide (2, 3). The severity of TB is dictated by the outcome of the battle between M. tuberculosis and the host immune system. However, M. tuberculosis has evolved numerous strategies to hijack the host immune response to favor its intracellular survival (4–6). This balance is further tipped in favor of M. tuberculosis by the growing emergence of multidrug-resistant tuberculosis (MDR-TB) and extensively drug-resistant TB (XDR-TB) strains, rendering current anti-TB drugs ineffective (7). Further investigations to reveal the mechanisms underlying the interplay between M. tuberculosis and its host cells will undoubtedly provide valuable information for the development of more efficient and selective therapeutic approaches against TB.

Histamine, which is generated from histidine by histidine decarboxylase (HDC) (8), promotes various physiological functions, including cell proliferation, differentiation, and embryonic development (9, 10). In addition, it has been demonstrated that histamine not only triggers acute symptoms but also affects chronic inflammation and regulates several immune responses (8). However, reports of the roles of histamine in either proinflammatory or anti-inflammatory responses in different experimental systems are divergent, and even controversial, due to differences in the predominance of histamine receptors, the concentration of histamine, and the binding affinity between histamine and receptors (11, 12). To date, four transmembrane, G protein-coupled, histamine receptors, HRH1, HRH2, HRH3, and HRH4, have been identified, which mediate important cellular processes via signaling pathways activated by the ligand-receptor interaction (8, 11). HRH1 is expressed ubiquitously in human mast cells, dendritic cells, immune cells, and many other cell types, and is mainly involved in allergy and inflammation. Investigations on HRH1-deficient mice have indicated that the release of IL-4 and IL-13 is increased, and lung allergic responses are attenuated, suggesting that HRH1 enhances Th2-type immune responses (13).

Recent reports on the crosstalk between histamine and M. tuberculosis are contradictory. Investigations showed that H37Rv infection significantly enhanced histamine levels in C57BL/6 mice at day 28 postinfection, and augmented the levels of IL-12, interferon-gamma, and nitric oxide as well as the numbers of activated CD4^+^T cells and CD11c^+^ cells in the lungs of infected HDC^−/−^ mice, indicating that histamine functions as a regulator of the host response against M. tuberculosis infection (14). In contrast, another study showed that HDC^−/−^ macrophages infected with BCG produced lower levels of IL-18 with enhanced mycobacterial titers, suggesting that histamine activates the BCG-induced IL-18 production process via the HRH1 and HRH2 signaling pathways (15). Thus, the mechanism underlying the role of histamine receptors in M. tuberculosis infection remains to be fully elucidated (16). In the present study, we demonstrated that M. tuberculosis utilizes HRH1 to inhibit the GRK2-p38MAPK signaling pathway, and thus decrease p47^phox^ phosphorylation levels, leading to NOX2 inactivation, reduced cROS production, and phagosome maturation.

## RESULTS

### Histamine-activated HRH1 signaling enhanced the intracellular survival of M. tuberculosis in macrophages.

**FIG 1:**
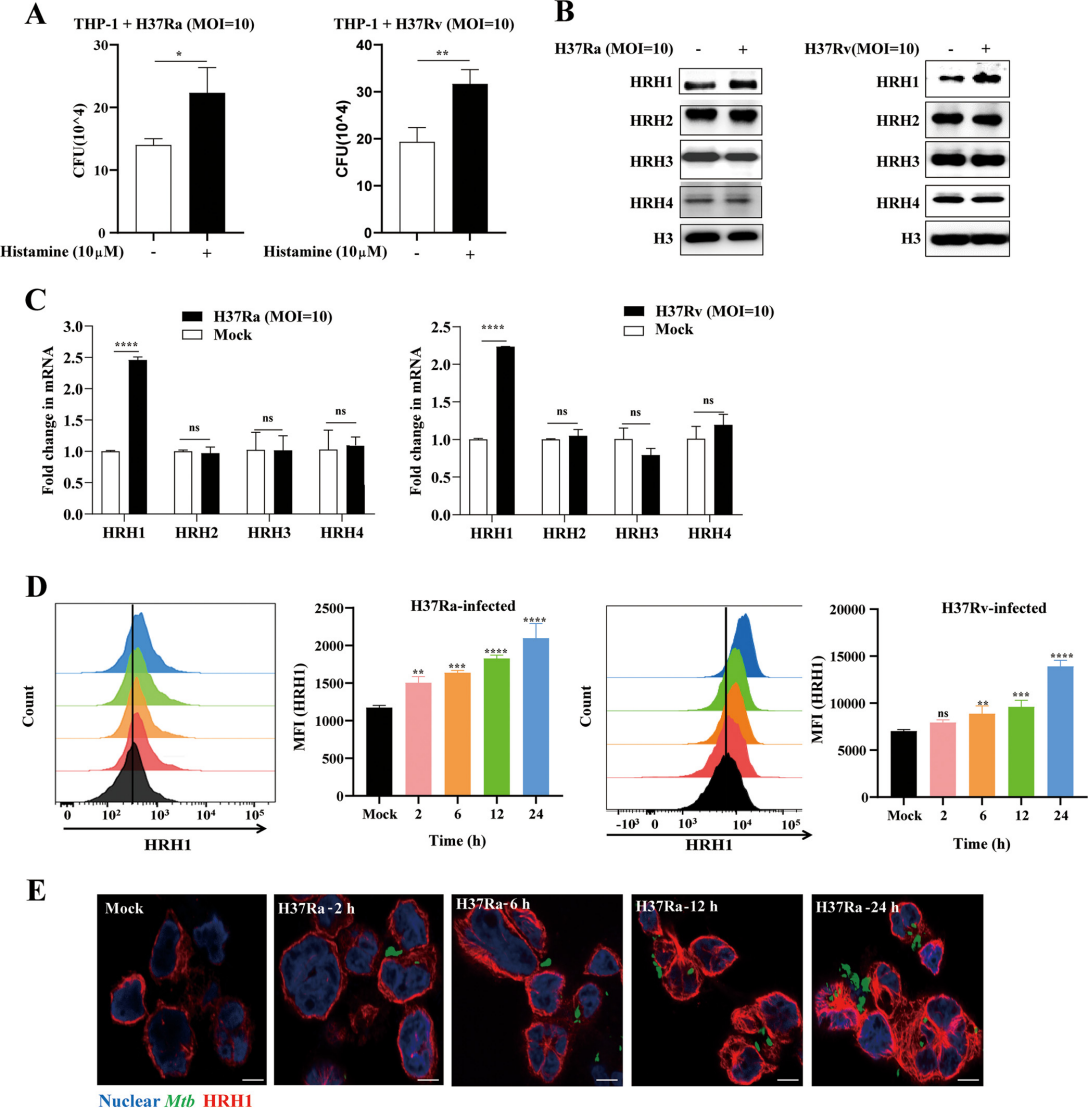
Histamine-activated HRH1 signaling enhanced the intracellular survival of M. tuberculosis in macrophages. (A) Colony forming unit (CFU) analysis of intracellular mycobacteria in PMA-differentiated THP-1 macrophages infected with H37Ra or H37Rv (MOI = 10:1) for 6 h, and then treated with 10 μM histamine for another 72 h. (B) Western blotting of protein expression levels of the four histamine receptors in THP-1 macrophages infected with H37Ra or H37Rv (MOI = 10:1) for 24 h. (C) RT-qPCR analysis of THP-1 macrophages infected with H37Ra or H37Rv (MOI = 10:1) for 24 h. (D) Flow cytometric analysis of HRH1 expression in THP-1 macrophages infected with H37Ra or H37Rv (MOI = 10:1); data represent HRH1^+^ cell counts and HRH1 mean fluorescence intensity (MFI). (E) Representative immunofluorescence confocal microscopy images (total 80 to 100 images, scale bars: 5 μm) of HRH1 expression in THP-1 macrophages infected with GFP labeled H37Ra (MOI = 10:1). All data represent the mean ± SD of at least three experiments. *, *P < *0.05; **, *P < *0.01; ***, *P < *0.001; ****, *P < *0.0001; ns, not significant.

Previous studies suggested that M. tuberculosis infection boosts the production of histamine in mice (14) and rat mast cells (17). However, the role of histamine in the intracellular survival of mycobacteria is unclear (14, 15). In the present study, we first explored the effects of histamine on the intracellular survival of mycobacteria in macrophages. Our data suggested that the survival of H37Ra or H37Rv in THP-1 cells was enhanced (*P* < 0.05) following the addition of exogenous histamine (10 μM) (Fig. 1A). And the bactericidal or cytotoxic effect of 10 μM histamine on H37Ra or THP-1 differentiated macrophages was excluded, respectively (Fig. S1A and 1B).

10.1128/mbio.02004-22.1 FIG S1 The effect of histamine on mycobacteria and macrophages. (A) Growth rates of H37Ra in 7H9 medium containing histamine; DMSO was used as solvent control. (B) Cell viability analysis of THP-1 treated with different concentrations of histamine for 24 h. All data represent the mean ± SD of at least three experiments. *, *P < *0.05. Download FIG S1, JPG file, 2.0 MB. Copyright © 2022 Mo et al. 2022 Mo et al. https://creativecommons.org/licenses/by/4.0/ This content is distributed under the terms of the Creative Commons Attribution 4.0 International license.

To determine which of the four major histamine receptors mediate the enhanced survival of mycobacteria in macrophages, we measured the expression HRH1 to HRH4 in THP-1 cells infected with avirulent H37Ra or virulent H37Rv. Surprisingly, only HRH1 was significantly (*P* < 0.0001) upregulated at both the protein and mRNA levels in THP-1 macrophages infected with either H37Ra or H37Rv, with no significant changes in the expression of the other three types of histamine receptors (HRH2 to 4) (Fig. 1B and C). The enhanced expression of HRH1 in THP-1 cells infected with H37Ra or H37Rv was further confirmed by flow cytometry (Fig. 1D) and immunofluorescence confocal microscopy (Fig. 1E).

Collectively, our findings suggested that histamine was beneficial for the survival of M. tuberculosis in macrophages, and the expression of HRH1, but not other HRHs, was upregulated by M. tuberculosis, indicating that histamine played a protective role in the survival of M. tuberculosis in macrophages via HRH1 signaling.

### Inhibition or knockdown of HRH1 attenuated M. tuberculosis survival in macrophages.

**FIG 2:**
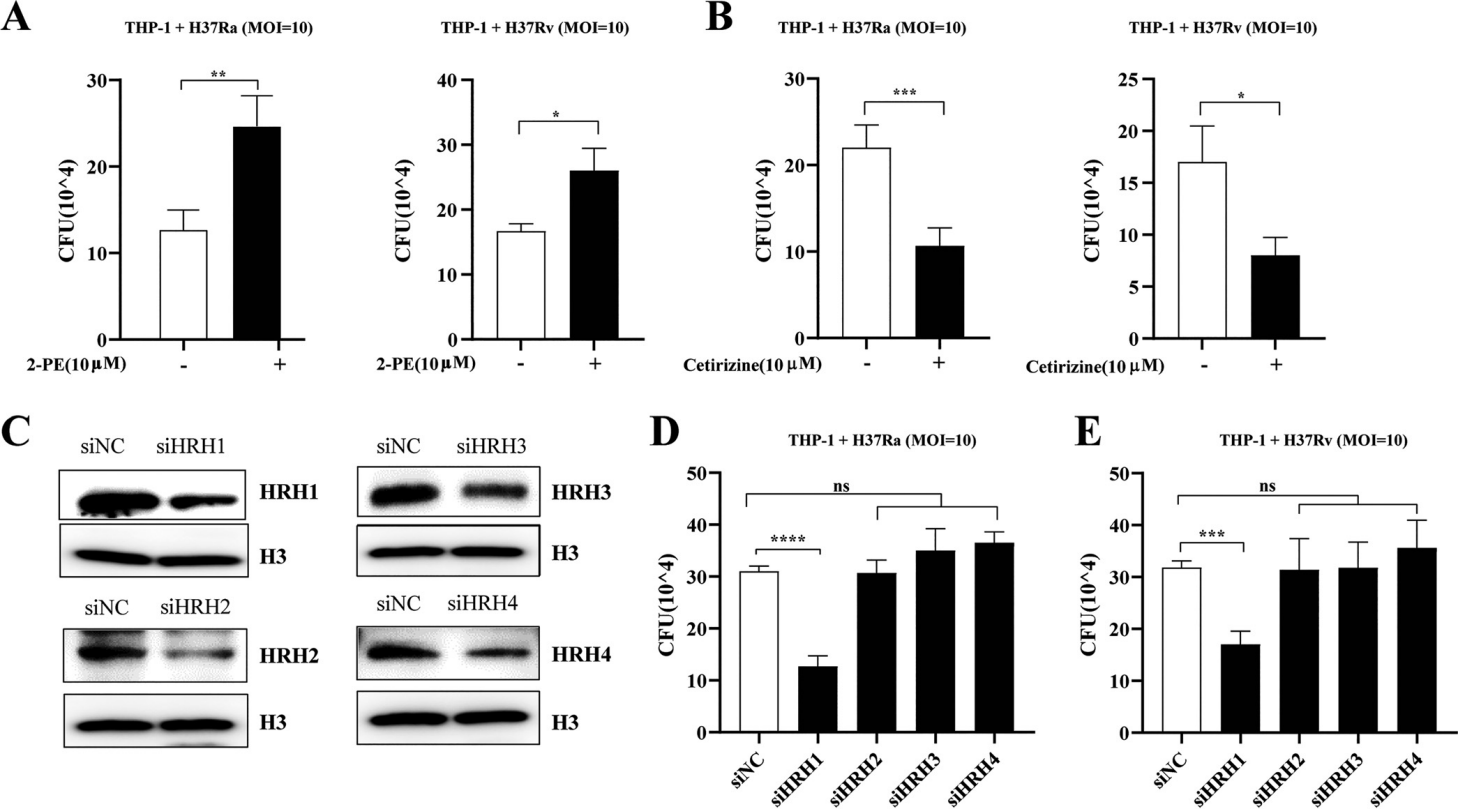
Inhibition or knockdown of HRH1 attenuated M. tuberculosis survival in macrophages. (A) Colony forming unit (CFU) analysis of intracellular mycobacteria in PMA-differentiated THP-1 macrophages infected with H37Ra or H37Rv for 6 h, and then treated with 10 μM 2-PE (HRH1 agonist) for 72 h. (B) Colony forming unit (CFU) analysis of intracellular mycobacteria in PMA-differentiated THP-1 macrophages infected with H37Ra or H37Rv (MOI = 10:1) for 6 h before treatment with 10 μM cetirizine (HRH1 antagonist) for 72 h. (C) Western blotting of histamine receptor (HRH1 to 4) protein expression level in THP-1 cells transfected for 48 h with gene-targeted siRNAs. (D) Colony forming unit (CFU) analysis of intracellular mycobacteria in PMA-differentiated THP-1 macrophages transfected for 48 h with gene-targeted siRNAs before infection with H37Ra (MOI = 10:1) for 72 h. (E) Colony forming unit (CFU) analysis of intracellular mycobacteria in PMA-differentiated THP-1 macrophages transfected for 48 h with gene-targeted siRNAs before infection with H37Rv (MOI = 10:1) for 72 h. All data represent the mean ± SD of at least three experiments. *, *P < *0.05; **, *P < *0.01; ***, *P < *0.001; ****, *P < *0.0001; ns, not significant.

To test the hypothesis that M. tuberculosis survival in macrophages was enhanced via host HRH1, we treated mycobacteria-infected macrophages with the HRH1 agonist 2-PE or the HRH1 antagonist cetirizine, both of which were confirmed to have no significant effects on the growth of mycobacteria or the viability THP-1 cells at concentrations lower than 10 μM (Fig. S2A to D). We found that the survival of mycobacteria in macrophages was enhanced by the HRH1 agonist 2-PE (Fig. 2A) but abolished by the HRH1 antagonist cetirizine (Fig. 2B). After confirming the effectiveness of siRNA-mediated HRH knockdown in THP-1 cells (Fig. 2C), we then analyzed the survival of mycobacteria in HRH-silenced macrophages. In accordance with our previous findings, downregulation of HRH1, but not the other three HRHs, was detrimental to the survival of both H37Ra (*P < *0.0001) and H37Rv (*P < *0.001) in macrophages (Fig. 2D and E). Taken together, our data demonstrated that M. tuberculosis exploited the host HRH1 to favor its intracellular survival in macrophages.

10.1128/mbio.02004-22.2 FIG S2 The effect of 2-PE or cetirizine on mycobacteria and macrophage growth and viability. (A and B) Growth curves of H37Ra cultured in a 7H9 medium containing various concentrations of 2-PE or cetirizine; DMSO was used as solvent control. (C and D) Viability of THP-1 cells cultured in the presence of 2-PE or cetirizine for 24 h. All data represent the mean ± SD of at least three experiments. **, *P < *0.01; ***, *P < *0.001. Download FIG S2, JPG file, 2.5 MB. Copyright © 2022 Mo et al. 2022 Mo et al. https://creativecommons.org/licenses/by/4.0/ This content is distributed under the terms of the Creative Commons Attribution 4.0 International license.

### HRH1 suppressed NOX2-mediated cROS production.

**FIG 3:**
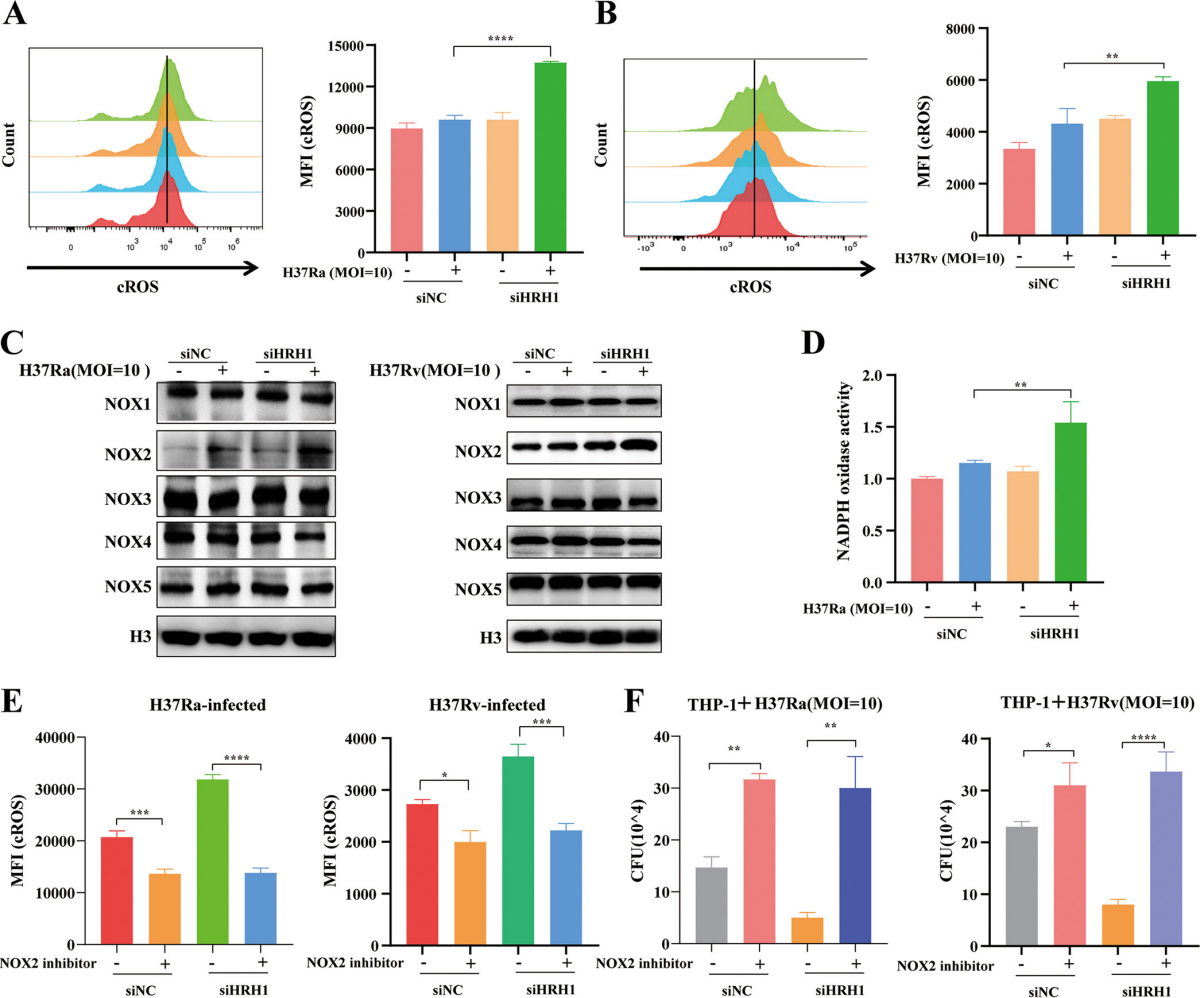
HRH1 suppressed NOX2-mediated cROS production. (A) PMA-differentiated THP-1 macrophages were transfected with HRH1 or control siRNA for 48 h before infection with H37Ra (MOI = 10:1) for 24 h. Cytoplasmic ROS (cROS) was analyzed by flow cytometry. (B) PMA-differentiated THP-1 macrophages were transfected with HRH1 or control siRNA for 48 h before infection with H37Rv (MOI = 10:1) for 24 h. Cytoplasmic ROS (cROS) was analyzed by flow cytometry. (C) Western blotting of the protein expression level of NOX family members following either infection with H37Ra and H37Rv alone or in combination and with or without HRH1 knockdown. (D) PMA-differentiated THP-1 macrophages were transfected with HRH1 or control siRNA for 48 h before infection with H37Ra (MOI = 10:1) for 24 h. NADPH oxidase activity was analyzed by measuring the absorbance at 450 nm using the EpochTM 2 microplate spectrophotometer. (E) Flow cytometric analysis of cROS expression following infection with H37Ra or H37Rv and in the presence or absence of 25 μM NOX2 inhibitor. (F) Colony forming unit (CFU) analysis of intracellular mycobacteria in THP-1 macrophages with or without 25 μM NOX2 inhibitor. All data represent the mean ± SD of at least three experiments. *, *P < *0.05; **, *P < *0.01; ***, *P < *0.001; ****, *P < *0.0001; ns, not significant.

Previous studies suggested that histamine induces reactive oxygen species (ROS) production via its receptors to modulate the activity of microglia or whole blood phagocytes (18, 19). Therefore, we next investigated the crosstalk between HRH1 and ROS production in macrophages. Flow cytometric analysis indicated that in the presence of HRH1, H37Ra or H37Rv infection did not induce significant production of cytosolic ROS (cROS), whereas cROS production was enhanced by mycobacterial infection in the absence of HRH1 (Fig. 3A and B). However, mitochondrial ROS (mROS) production remained stable under the same conditions (Fig. S3A and B). These data suggested that HRH1 suppressed the cROS production in macrophages exposed to M. tuberculosis infection.

10.1128/mbio.02004-22.3 FIG S3 The role of HRH1 on mROS production, autophagy, and apoptosis of mycobacteria-infected macrophages. (A) PMA-differentiated THP-1 macrophages were transfected with HRH1 or control siRNA for 48 h and then infected with H37Ra (MOI = 10:1) for 24 h. Mitochondrial ROS (mROS) production was analyzed by flow cytometry. (B) PMA-differentiated THP-1 macrophages were transfected with HRH1 or control siRNA for 48 h and then infected with H37Rv (MOI = 10:1) for 24 h. Mitochondrial ROS (mROS) production was analyzed by flow cytometry. (C) PMA-differentiated THP-1 macrophages were transfected with HRH1 or control siRNA for 48 h and then infected with H37Ra (MOI = 10:1) for 24 h. The protein concentration was determined using a BCA assay kit. Total protein levels in all samples were standardized by comparison with the control sample (the red column). (D) Western blotting of LC3-1 and LC3-II (autophagy markers) protein levels. (E) PMA-differentiated THP-1 macrophages were transfected with HRH1 or control siRNA for 48 h and then infected with H37Ra (MOI = 10:1) for 24 h. Apoptotic cells were then analyzed by flow cytometry. All data represent the mean ± SD of at least three experiments. ns, not significant. Download FIG S3, JPG file, 2.0 MB. Copyright © 2022 Mo et al. 2022 Mo et al. https://creativecommons.org/licenses/by/4.0/ This content is distributed under the terms of the Creative Commons Attribution 4.0 International license.

To elucidate the mechanism by which HRH1 suppresses cROS production, we focused on the NADPH oxidase (NOX) family due to their biological function in generating ROS (20). Interestingly, H37Ra or H37Rv infection enhanced NOX2 expression in THP-1 cells, and the expression of NOX2 was further elevated following siRNA-mediated HRH1 knockdown but had no significant effects on the expression of other NOX family members in macrophages under the same conditions (Fig. 3C). Moreover, we found that HRH1 inhibited NADPH oxidase activity as well (Fig. 3D and Fig. S3C). These findings suggested that HRH1 played a negative regulatory role in both the expression and activity of NOX2. In addition, flow cytometric analysis confirmed that cROS production was also significantly suppressed in H37Ra or H37Rv infected macrophages following treatment with the NOX2-specific inhibitor GSK2795039 (Fig. 3E). Similarly, the mycobacterial burden in infected macrophages was significantly (*P < *0.01) enhanced under the same treatment conditions (Fig. 3F). These data indicated that cROS production in mycobacteria-infected macrophages was modulated via the HRH1-NOX2 axis.

A previous study indicated an interplay between ROS and autophagy (21). In our experimental system, we analyzed the expression of LC3-1 and LC3-II as markers of autophagy and showed that THP-1 autophagy was enhanced ≥12 h after M. tuberculosis infection, while siRNA-mediated HRH1 knockdown had no synergetic effect on autophagy after mycobacterial infection (Fig. S3D). Furthermore, flow cytometric analysis confirmed that THP-1 apoptosis was enhanced following M. tuberculosis infection, and this was not affected by siRNA-mediated HRH1 knockdown (Fig. S3E).

Thus, our findings suggested that M. tuberculosis-induced upregulation of HRH1 expression suppressed the expression and activity of NOX2, and subsequently inhibited cROS production to benefit the survival of mycobacteria in macrophages.

### M. tuberculosis infection inhibited phagosome maturation and acidification via the HRH1-NOX2 axis.

**FIG 4:**
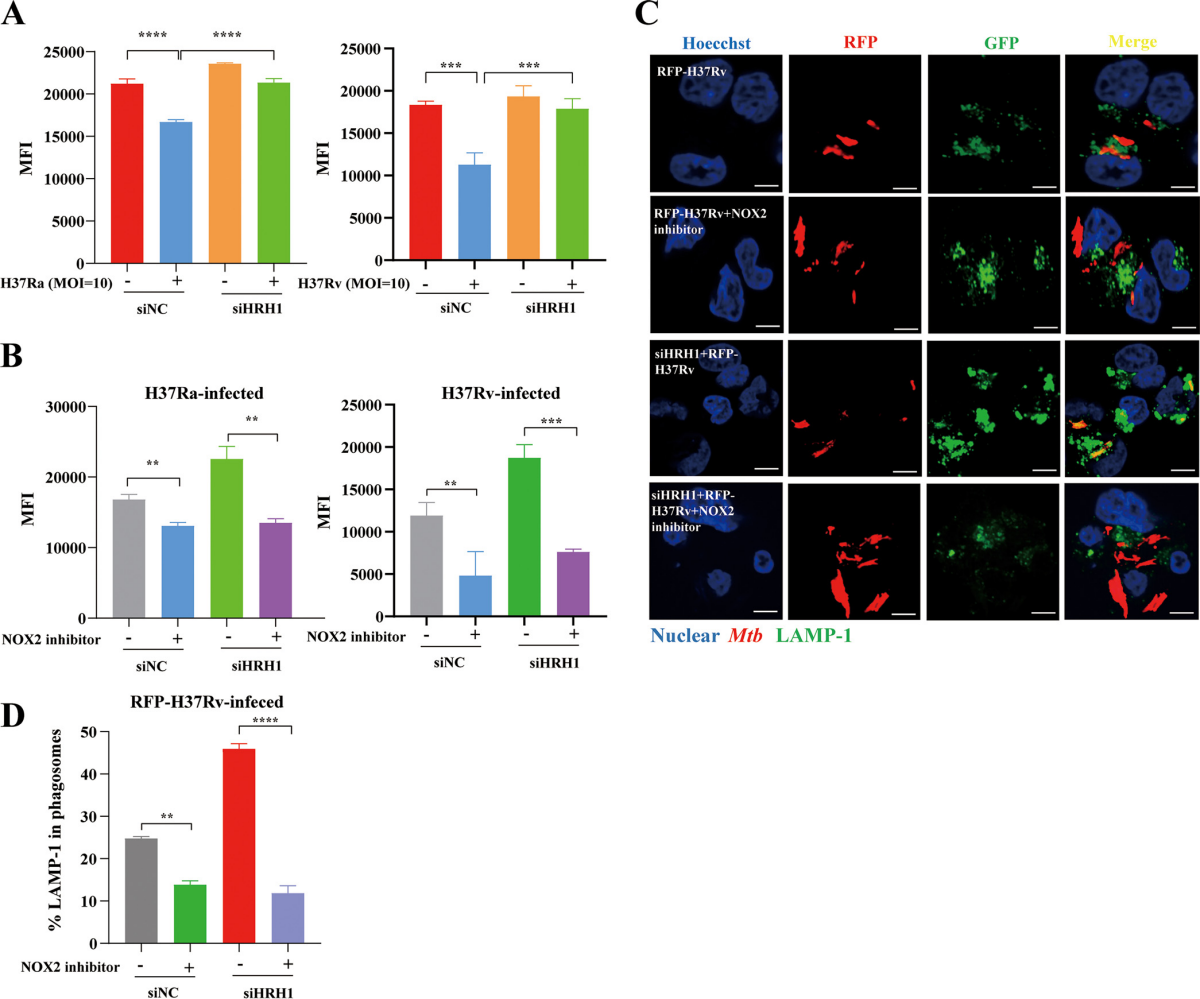
M. tuberculosis infection inhibits phagosome maturation and acidification via the HRH1-NOX2 axis. (A) PMA-differentiated THP-1 macrophages were transfected with HRH1 or control siRNA for 48 h before infection with H37Ra or H37Rv (MOI = 10:1) for another 24 h. Flow cytometric analysis of intracellular phagosome acidification levels. (B) PMA-differentiated THP-1 macrophages were transfected with HRH1 or control siRNA for 48 h before infection with H37Ra or H37Rv (MOI = 10:1) and cultured with or without the NOX2 inhibitor (25 μM) for another 24 h. Flow cytometric analysis of intracellular phagosome acidification levels. (C) Representative immunofluorescence confocal microscopy images (total 80 to 100 images) to illustrate the staining of phagosome maturation in RFP-H37Rv infection macrophages; scale bars: 5 μm. (D) Quantification of phagosome maturation using LAMP-1 as an indicator. All data represent the mean ± SD of at least three experiments. **, *P < *0.01; ***, *P < *0.001; ****, *P < *0.0001; ns, not significant.

A previous study suggested that NOX2 regulates phagosomal pH in dendritic cells during antigen processing (22). In this study, we investigated the crosstalk between HRH1 and NOX2 in the process of phagosome maturation and acidification. Our data suggested that mycobacterial infection inhibited phagosome acidification, and this effect was abolished following siRNA-mediated HRH1 knockdown (Fig. 4A), indicating the inhibitory effect of HRH1 on the phagosome acidification process. Phagosomal acidification was also blocked by the NOX2-specific inhibitor GSK2795039 (Fig. 4B). Lysosome-associated membrane protein 1 (LAMP-1) is delivered to phagosomes during the maturation and acidification process (23). Following fluorescently labeled H37Ra or H37Rv infection, HRH1 suppressed the fusion of phagosomes and lysosomes. Furthermore, inhibitory effects of HRH1 on phagosome maturation and acidification were counteracted in the presence of the NOX2 inhibitor (Fig. 4B to D and Fig. S4). Taken together, our findings indicated that M. tuberculosis utilized the host HRH1 to modulate the expression and activity of NOX2, which played a critical role in generating ROS and promoting phagosome maturation and acidification.

10.1128/mbio.02004-22.4 FIG S4 M. tuberculosis infection inhibited phagosome maturation and acidification via the HRH1-NOX2 axis. PMA-differentiated THP-1 macrophages were transfected with HRH1 or control siRNA for 48 h and then infected with GFP-H37Ra (MOI = 10:1) for another 24 h. (A) Representative immunofluorescence confocal microscopy images (total 80 to 100 images) to illustrate the staining of phagosome maturation in GFP-H37Ra infected macrophages; scale bars: 5 μm. (B) Quantification of phagosome maturation using LAMP-1 as an indicator. All data represent the mean ± SD of at least three experiments. *, *P < *0.05; **, *P < *0.01. Download FIG S4, JPG file, 1.7 MB. Copyright © 2022 Mo et al. 2022 Mo et al. https://creativecommons.org/licenses/by/4.0/ This content is distributed under the terms of the Creative Commons Attribution 4.0 International license.

### HRH1 inhibited p47^phox^ phosphorylation to suppress NOX2 activity via the p38MAPK signaling pathway.

**FIG 5:**
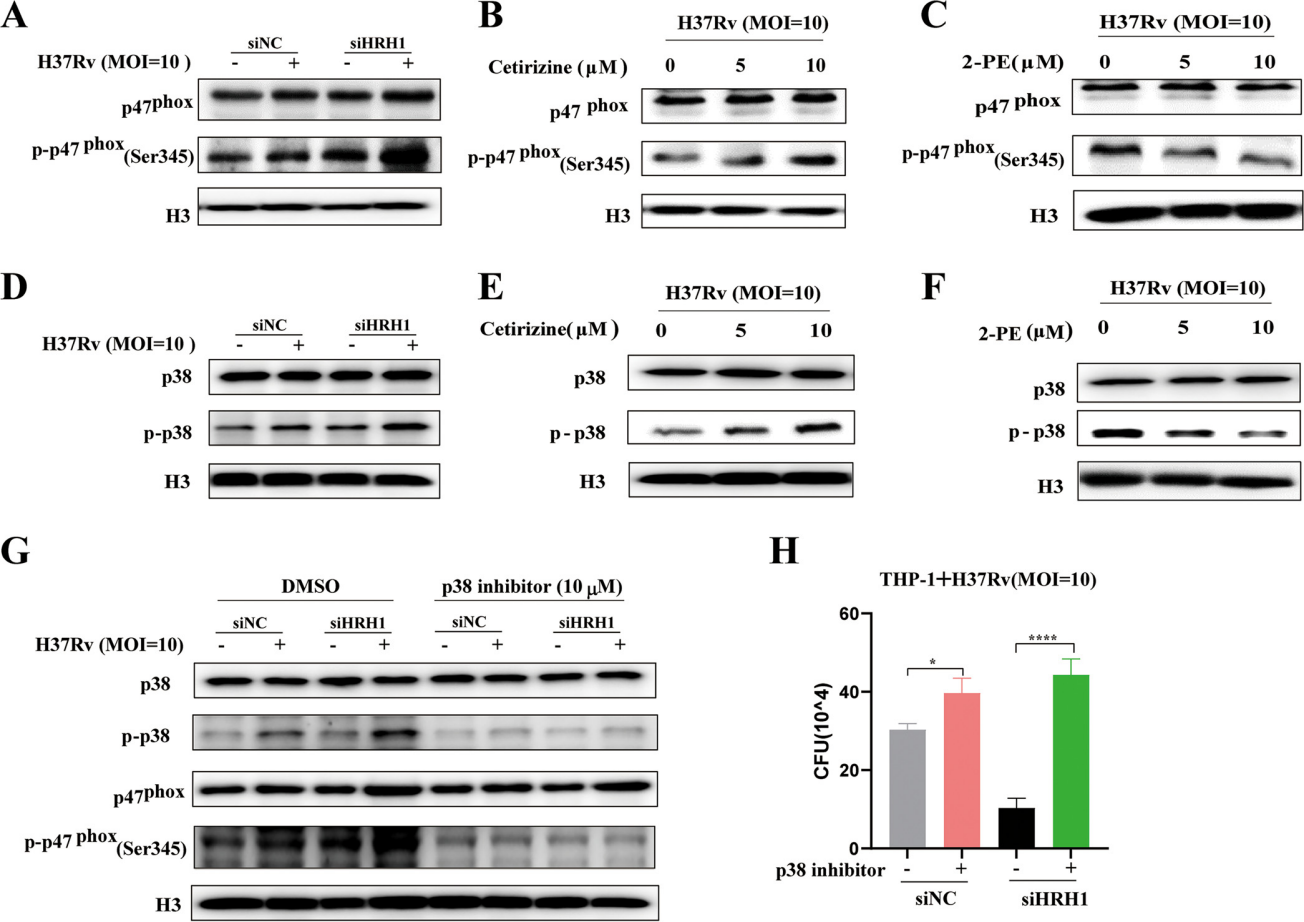
HRH1 inhibited p47^phox^ phosphorylation to suppress NOX2 activity via the p38MAPK signaling pathway. (A) Western blotting of the protein levels of p47^phox^ and p-p47^phox^ (Ser345) in PMA-differentiated THP-1 cells transfected with HRH1 or control siRNA for 48 h before infection with H37Rv (MOI = 10:1) for another 24 h. (B) Western blotting of the protein levels of p47^phox^ and p-p47^phox^ (Ser345) in the presence or absence of cetirizine (0 μM, 5 μM, 10 μM). (C) Western blotting of the protein levels of p47^phox^ and p-p47^phox^ (Ser345) in the presence or absence of 2-PE (0 μM, 5 μM, 10 μM). (D) Western blotting of the protein levels of p38MAPK and p-p38MAPK in PMA-differentiated THP-1 cells transfected with HRH1 or control siRNA for 48 h before infection with H37Rv (MOI **=** 10:1) for another 24 h. (E) Western blotting of the protein levels of p38MAPK and p-p38MAPK in PMA-differentiated THP-1 cells transfected with HRH1 or control siRNA for 48 h and cultured in the presence or absence of cetirizine (0 μM, 5 μM, 10 μM). (F) Western blotting of the protein levels of p38MAPK and p-p38MAPK in the presence or absence of 2-PE (0 μM, 5 μM, 10 μM). (G-H) PMA-differentiated THP-1 cells were transfected with HRH1 or control siRNA for 48 h before infection with H37Rv (MOI **=** 10:1) for another 24 h in the presence or absence of 10 μM p-p38MAPK inhibitor. (G) Western blotting of the protein levels of p38MAPK, p-p38MAPK, p47^phox^, and p-p47^phox^ (Ser345). (H) Colony forming unit (CFU) analysis of intracellular bacteria. All data represent the mean ± SD of at least three experiments. *, *P < *0.05; ****, *P < *0.0001.

During the process of phagocytosis, NOX2 is assembled in phagosomes and activated via the phosphorylation of p47^phox^ (Ser345) (22, 24). To elucidate the mechanism by which HRH1 regulates the function of NOX2, we compared the phosphorylation levels of p47^phox^ (Ser345) under various conditions. Our data indicated that the total p47^phox^ and phosphorylated p47^phox^ protein levels were slightly changed following H37Rv or H37Ra infection. However, the level of phosphorylated p47^phox^ (Ser345) was enhanced following siRNA-mediated HRH1 knockdown and was further elevated by mycobacterial infection (Fig. 5A and Fig. S5A). Furthermore, we showed that the levels of phosphorylated p47^phox^ (Ser345) were elevated in the presence of the HRH1 antagonist cetirizine and abolished by the agonist 2-PE. The effects of both drugs on p47^phox^ (Ser345) phosphorylation were dose-dependent (Fig. 5B and C; Fig. S5B and C). These observations suggested that HRH1 modulated the phosphorylation of p47^phox^ (Ser345).

10.1128/mbio.02004-22.5 FIG S5 HRH1 inhibited p47^phox^ phosphorylation to suppress NOX2 activity via the p38MAPK signaling pathway. (A) Western blotting of the protein levels of p47^phox^ and p-p47^phox^ in PMA-differentiated THP-1 cells transfected with HRH1 or control siRNA for 48 h and then infected with H37Ra (MOI **=** 10:1) for another 24 h. (B) Western blotting of the protein levels of p47^phox^ and p-p47^phox^ in the presence or absence of cetirizine (0 μM, 5 μM, 10 μM). (C) Western blotting of the protein levels of p47^phox^ and p-p47^phox^ in the presence or absence of 2-PE (0 μM, 5 μM, 10 μM). (D) Western blotting of the protein levels of p38MAPK and p-p38MAPK in PMA-differentiated THP-1 cells transfected with HRH1 or control siRNA for 48 h and then infected with H37Ra (MOI **=** 10:1) for another 24 h. (E) Western blotting of the protein levels of p38MAPK and p-p38MAPK in PMA-differentiated THP-1 cells modified as described in (D) and cultured in the presence or absence of cetirizine (0 μM, 5 μM, 10 μM). (F) Western blotting of the protein levels of p38MAPK and p-p38MAPK in PMA-differentiated THP-1 cells modified as described in (D) and cultured in the presence or absence of 2-PE (0 μM, 5 μM, 10 μM). (G-H) PMA-differentiated THP-1 cells were transfected with HRH1 or control siRNA for 48 h and infected with H37Ra (MOI **=** 10:1) for 24 h in the presence or absence of 10 μM p-p38MAPK inhibitor. (G) Western blotting of the protein levels of p38MAPK, p-p38MAPK, p47^phox^, and p-p47^phox^. (H) CFU of intracellular bacteria. All data represent the mean ±SD of at least three experiments. ***, *P < *0.001. Download FIG S5, JPG file, 2.0 MB. Copyright © 2022 Mo et al. 2022 Mo et al. https://creativecommons.org/licenses/by/4.0/ This content is distributed under the terms of the Creative Commons Attribution 4.0 International license.

The p38 mitogen-activated protein kinase (MAPK) signaling pathway is activated by different intracellular stimuli and a wide variety of environmental stresses, such as pathogen infection (25). Inhibition of p38MAPK has been reported to abolish the phosphorylation of p47^phox^ induced by the selective TLR8 agonist CL075, suggesting crosstalk between p38MAPK and p47^phox^ phosphorylation and NOX2 activation (26). Similar to the phosphorylation pattern of p47^phox^ (Ser345), the p38MAPK phosphorylation level was augmented following mycobacterial infection, and further enhanced in the absence of HRH1 (Fig. 5D and Fig. S5D). Furthermore, phosphorylated p38MAPK levels were elevated in the presence of the HRH1 antagonist cetirizine and abolished by the agonist 2-PE. The effects of both drugs on p38 MAPK phosphorylation were also dose-dependent (Fig. 5E and F; Fig. S5E and F). These findings indicated that HRH1 played an inhibitory role on p38MAPK phosphorylation. In addition, treatment of the macrophages with Doramapimod, a highly potent p38MAPK inhibitor, counteracted the effects of mycobacterial infection and HRH1 on p47^phox^ phosphorylation (Fig. 5G and Fig. S5G). The bacterial burden in infected macrophages was also significantly (*P < *0.001) increased after p38MAPK inhibitor treatment (Fig. 5H and Fig. S5H). These data indicated that HRH1 inactivated NOX2 activity via the p38MAPK signaling pathway.

### HRH1 and GRK2 interacted to negatively regulate p38MAPK signaling.

**FIG 6:**
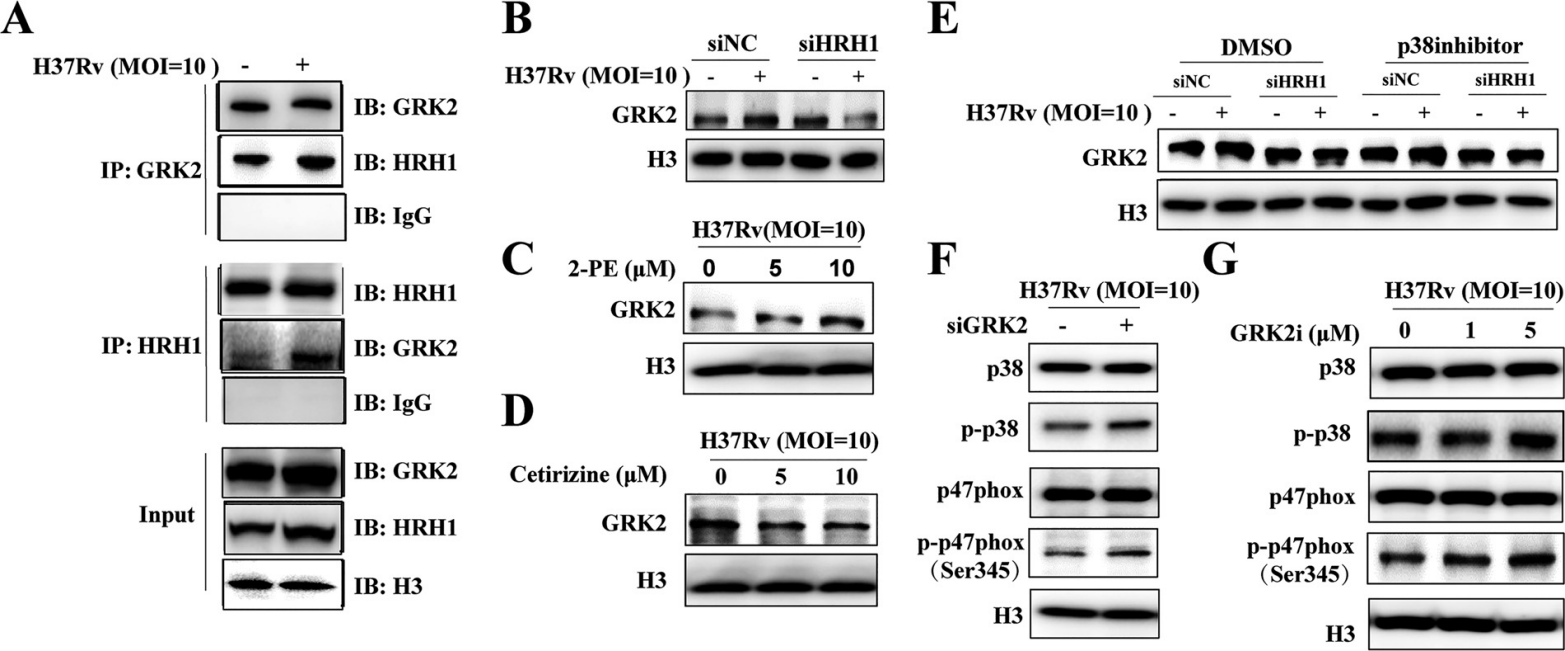
HRH1 interacts with GRK2 to negatively regulate the p38MAPK signaling pathway. (A) PMA-differentiated THP-1 cells were infected with H37Rv (MOI = 10:1) for 24 h and immunoprecipitated (IP) with anti-GRK2 or anti-HRH1 antibodies. Immunoprecipitates were then immunoblotted (IB) with anti-GRK2 or anti-HRH1 antibodies, accordingly. (B) Western blotting of GRK2 protein levels in PMA-differentiated THP-1 cells transfected with HRH1 siRNA for 48 h and infected with H37Rv (MOI = 10:1) for 24 h. (C) Western blotting of GRK2 protein levels in the presence or absence of 2-PE (0, 5 μM, 10 μM) with H37Rv (MOI = 10:1) infection for 24 h. (D) Western blotting of GRK2 protein levels in the presence or absence of cetirizine (0, 5 μM, 10 μM) with H37Rv (MOI = 10:1) infection for 24 h. (E) Western blotting of GRK2 protein levels in PMA-differentiated THP-1 cells transfected with HRH1 siRNA for 48 h and infected with H37Rv (MOI = 10:1) for 24 h in the presence or absence of 10 μM p-p38MAPK inhibitor. (F) Western blotting of the protein levels of p47^phox^/p-p47^phox^ (Ser345), p38MAPK, and p-p38MAPK in PMA-differentiated THP-1 cells transfected with GRK2 siRNA for 48 h and infected with H37Rv (MOI = 10:1) for 24 h. (G) Western blotting of the protein levels of p47^phox^/p-p47^phox^ (Ser345), p38MAPK, and p-p38MAPK in the presence or absence of GRK2i (inhibitor, 0, 1, or 5 μM) with H37Rv (MOI = 10:1) infection for 24 h.

To elucidate the mechanism by which HRH1 regulates the p38MAPK signaling pathway, we investigated the crosstalk between HRH1 and the G protein-coupled receptor kinase type 2 (GRK2), which has been identified as a vital protein that regulates the transduction and trafficking of G protein-coupled receptors (GPCR) signaling (27). In addition, p38MAPK is negatively regulated by GRK2 (28). Interestingly, immunoprecipitation and Western blotting indicated a direct interaction between HRH1 and GRK2, especially under conditions of M. tuberculosis infection (Fig. 6A). GRK2 expression was elevated in H37Rv-infected macrophages and, importantly, this phenomenon was suppressed by the HRH1 silencing (Fig. 6B). Furthermore, we found that GRK2 expression levels were elevated in the presence of the HRH1 agonist 2-PE and abolished by the HRH1 antagonist cetirizine in a dose-dependent manner (Fig. 6C and D). Using the p38MAPK inhibitor Doramapimod, we then demonstrated that GRK2 was upstream of the p38MAPK signaling pathway (Fig. 6E). Furthermore, we then explored the ability of GRK2 to regulate p38MAPK by siRNA-mediated GRK2 knockdown or using GRK2 specific inhibitor GSK180736A. In the absence of GRK2, the phosphorylation level of p38MAPK and p47^phox^ was augmented following H37Rv infection; this effect was also induced by the GRK2 inhibitor in a dose-dependent manner (Fig. 6F and G). To conclude, our findings suggested that HRH1 interacted directly with GRK2 to negatively regulate the p38MAPK signaling pathway.

## DISCUSSION

**FIG 7:**
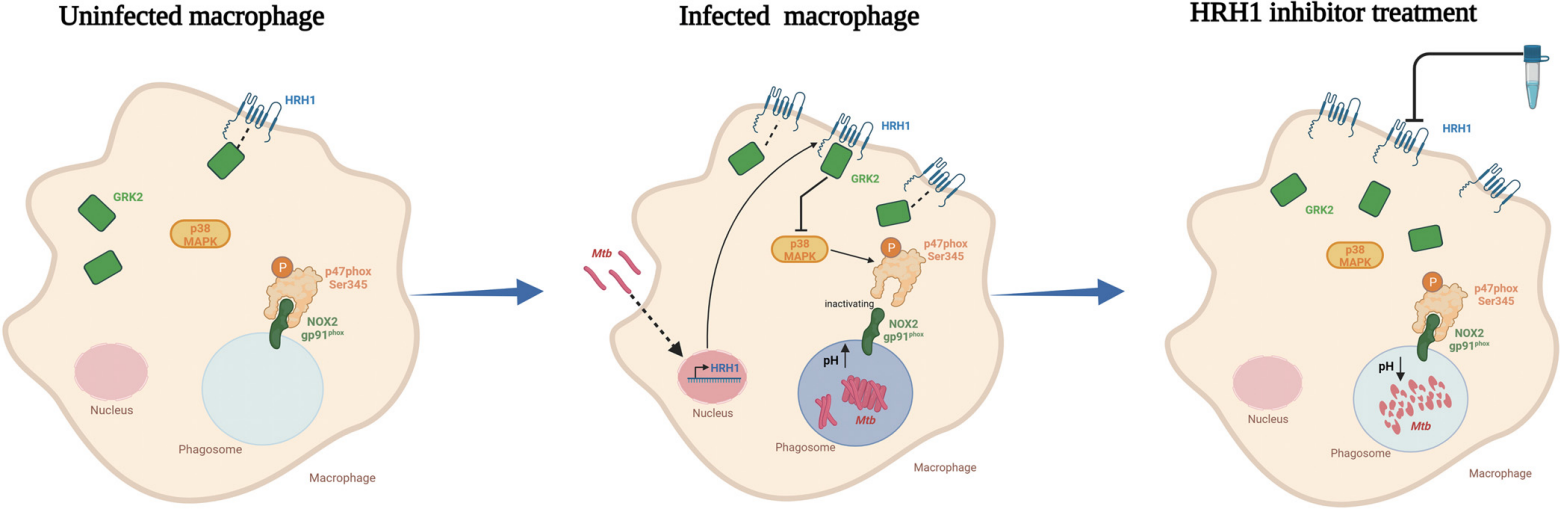
Mycobacterium tuberculosis utilized host HRH1 to modulate reactive oxygen species production and phagosome maturation via the GRK2-p38MAPK-NOX2 axis, a summary model. Uninfected macrophages displayed normal regulation of HRH1 and p38MAPK pathway (left). After mycobacteria infection and endocytosis by macrophages (middle panel), HRH1 expression was enhanced and interacted with GRK2, which then inhibited the p38MAPK signaling pathway. The inhibited p38MAPK signaling further decreased the level of phosphorylated oxidase subunit p47phox (Ser345), leading to the inability of the oxidase subunit p47^phox^ (Ser345) to bind NOX2 (gp91^phox^) on the phagosome membrane, and thus inhibited the NOX2 (gp91^phox^) oxidase complex formation. This reduced cROS production, which further prevented phagosome maturation and acidification. This process provided a beneficial environment that favored the intracellular survival of M. tuberculosis in macrophages. However, HRH1 inhibitor treatment blocked the inhibitory effect of GRK on p38MAPK signaling in M. tuberculosis-infected macrophages. As a result, NOX2 could function normally to promote phagosome maturation and acidification, which was detrimental to the survival of M. tuberculosis in macrophages (right).

M. tuberculosis is the most successful pathogen that coevolved with the host, and M. tuberculosis adopts various strategies to tackle the host's immune response to survive and replicate in the host's unfriendly environment. Targeting the vital process that M. tuberculosis crosstalk with the host may provide valuable information for the development of novel antituberculosis drugs. Histamine and its receptors were previously reported involve in the intracellular survival of M. tuberculosis in host macrophages, but the underlying mechanism remains to be elucidated. In the present study, we aimed to clarify the biological function of histamine and its receptors in M. tuberculosis-infected macrophages and elucidate the underlying mechanism. Our findings indicated that histamine was beneficial for the survival of M. tuberculosis in macrophages, and we provided evidence that the histamine receptor HRH1 played a vital role in suppressing cROS production, phagosome maturation, and acidification via the GRK2-p38MAPK-NOX2 signaling pathway (Fig. 7).

Histamine has been reported to function in numerous biological processes, such as inflammation and immune responses, via its four different receptors (8, 29). We found that histamine was beneficial for the survival of M. tuberculosis in macrophages via HRH1 signaling. In contrast to our findings, a previous study reported that exogenously added histamine decreased the intracellular counts of M. bovis BCG in HDC^−/−^ macrophages (15). This inconsistency may be attributed to a difference in the BCG and H37Rv used in the study, in addition to variations in the methods used to obtain murine bone-marrow macrophages and THP-1-derived macrophages, etc.

More importantly, the functions of histamine vary because of factors such as concentration and the predominance of these receptors on cells (11, 12). In inflammation-associated colonic tumorigenesis, histamine displayed a bimodal function, with a protumorigenic effect mediated via HRH1, while antitumorigenic effects were mediated by HRH2 (12). Ding et al. (30) recently reported that histamine acts via HRH1 to inhibit autophagy under conditions of hypoxia and acute myocardial infarction in cardiomyocytes of HDC^−/−^ mice. We did not detect the crosstalk between HRH1 and autophagy or apoptosis in our experimental system (Fig. S3). However, our data suggested that HRH1 inactivated NOX2, thereby reducing cROS production in M. tuberculosis-infected macrophages (Fig. 3). This difference in the functions of HRH1 may be due to the diverse downstream signaling pathways in different cells and mouse models, and further investigations are required to clarify this issue.

Histamine release by mast cells is significantly stimulated by M. tuberculosis infection (17), suggesting that the histamine biosynthesis or signaling pathways may be potential targets for novel anti-TB strategies. In the present study, we tested this hypothesis by treating M. tuberculosis-infected macrophages with the HRH1 antagonist cetirizine. We showed that this treatment attenuated mycobacterial loads in macrophages (Fig. 2B), which is consistent with a previous report (31). HRH1 and HRH2 have some similarities in terms of their expression in certain cell types and almost identical histamine binding domains (8). However, in the present study and other investigations, HRH1 was found to be the predominant mediator of mycobacterial survival in macrophages (14, 31). It can be speculated that the functions of HRH1 and HRH2 are determined by differences in factors such as histamine binding affinity for these two receptors and their abundance on cell surfaces.

Ji et al. (32) recently demonstrated that the HRH2 inhibitor roxatidine prevents fibrosis by inactivating the NF-κB and p38MAPK signaling pathways in macrophages. In addition, terfenadine, a selective chemical inhibitor of HRH1, was reported to enhance phosphor-p38MAPK levels in basal BC cells (33). In our investigations of crosstalk between HRH1 and the p38MAPK signaling pathway, we consistently showed that HRH1 inhibited p38MAPK signaling and then inactivated the NOX2 complex, thereby reducing cROS production in mycobacteria-infected macrophages (Fig. 7). Moreover, GRK2 is a ubiquitous member of the GRK family that appears to play a central, integrative role in signal transduction cascades, by participating together with arrestins in the regulation of GPCR (28). Based on this clue, we demonstrated that HRH1 interacted directly with GRK2 to negatively regulate the p38MAPK signaling pathway. To the best of our knowledge, the present study was the first to reveal the essential role of host HRH1 utilized by M. tuberculosis in its intracellular survival in macrophages and that this effect was mediated via the GRK2-p38MAPK and NOX2 signaling pathways. Further investigations are warranted to identify the M. tuberculosis virulence factor that mediates the interaction with host HRH1 and evaluate the effect of HRH1 activation on other immune cells, such as B and T cells.

In conclusion, we described the interaction between HRH1 and M. tuberculosis and clarified the underlying mechanism, which broadens our understanding of M. tuberculosis-host interaction and may provide useful information for the development of novel anti-TB strategies in the future.

## MATERIALS AND METHODS

### Reagents and inhibitors.

Histamine dihydrochloride (HY-B0722, MedChemExpress [MCE], USA), 2-pyridylethylamine dihydrochloride (2-PE) (APExBIO, USA), cetirizine (MCE, USA), and the p38 MAPK inhibitor Doramapimod (HY-10320, MCE, USA) were prepared as a 10 mM stock solution and diluted 1,000-fold for use in experiments. The NOX2 inhibitor GSK2795039 (HY-18950, MCE, USA) was prepared as a 10 mM stock solution and diluted to a working concentration of 25 μM. The GRK2 inhibitor GSK180736A (GRK2i, Selleck, S8489, USA) was prepared as a 100 mM stock solution and diluted to a working concentration of 10 μM. Unless stated otherwise, all reagents and inhibitors were prepared in DMSO (Sigma-Aldrich, Merck, USA). All stock solutions were stored at −80°C before use. Antibodies used in the present study are listed in Table S1.

10.1128/mbio.02004-22.6 TABLE S1 Antibodies used in this study. Download Table S1, DOC file, 0.04 MB. Copyright © 2022 Mo et al. 2022 Mo et al. https://creativecommons.org/licenses/by/4.0/ This content is distributed under the terms of the Creative Commons Attribution 4.0 International license.

### M. tuberculosis strains and growth.

H37Ra and H37Rv strains were cultured as previously described (34). Briefly, strains were inoculated in Difco Middlebrook 7H9 medium (BD, USA), supplemented with 10% oleic acid-albumin-glucose-catalase (OADC, BD, USA), 0.05% Tween 80 (Sigma-Aldrich, Merck, USA) and 0.2% glycerol (Sigma-Aldrich, Merck, USA), and cultured at 37°C with continuous shaking at 110 rpm. The mycobacteria were cultured to logarithmic growth phase (optical density at 600 nm [OD_600_] = 0.6 to 0.8) measured using an Epoch 2 microplate spectrophotometer (BioTek, USA), collected, and homogenized in a fresh 7H9 medium containing 15% (vol/vol) glycerol, and stored at −80°C. Before cell infection, the bacterial density was further confirmed by CFU analysis (CFU) on Difco Middlebrook 7H10 agar plates supplemented with 10% OADC and 0.5% glycerol.

### Growth curve evaluation.

The growth pattern of H37Ra in the presence or absence of histamine or other compounds was analyzed according to a modified version of a previously described protocol (35). Briefly, frozen H37Ra was first inoculated into 10 mL Middlebrook 7H9 broth supplemented with 10% OADC, 0.05% Tween 80, and 0.2% glycerol, and cultured to mid-log-phase measured using the Epoch^TM^ 2 microplate spectrophotometer. H37Ra was further diluted and subcultured in 50 mL of the same medium, with a starting OD_600_ value of 0.05, and cultured at 37°C with continuous shaking at 110 rpm. Histamine dihydrochloride (histamine), 2-PE, and cetirizine were serially diluted and added to cultures to evaluate their effect on H37Ra growth. H37Ra was cultured without drug or DMSO as positive and negative controls, respectively. The growth and survival of H37Ra under different conditions were assayed on alternate days using an Epoch^TM^ 2 microplate spectrophotometer. All tests were performed in triplicate.

### Cell culture.

The human monocytic cell line THP-1 was purchased from the Cell Bank of the Chinese Academy of Sciences (Shanghai, China) and cultured in RPMI 1640 medium (Corning, USA) supplemented with l-glutamine (2 mM) and 10% fetal bovine serum (Gibco, Life Technologies). For macrophage differentiation, THP-1 cells were seeded in 12-well plates (4 × 10^5^ cells/mL) and treated with 20 ng/mL phorbol 12-myristate 13-acetate (PMA; Sigma-Aldrich, Merck, USA) for 24 h at 37°C in a humidified atmosphere containing 5% CO_2_. Differentiated THP-1 cells were maintained under the same conditions in fresh complete RPMI 1640 medium for subsequent experiments.

### Cell viability.

Differentiated THP-1 cells were treated with histamine, 2-PE, or cetirizine at appropriate concentrations for 24 h in 96-well plates (5 × 10^3^ cells/well); cells treated with DMSO were included as a negative control. After treatment, WST-1 reagent (Beyotime, Shanghai, China) was added (10 μL/well) and the cells were incubated for 2 h at 37°C. The formazan dye produced from WST-1 by viable cells was quantified by measuring the absorbance at 450 nm using the Epoch^TM^ 2 microplate spectrophotometer. The absorbance at 650 nm was also measured as the reference wavelength. Cell viability was calculated according to the manufacturer’s instructions.

### Small interfering RNA transfection.

Target gene-specific siRNAs and negative control siRNA (NC), were synthesized and diluted with distilled water as instructed. PMA-treated THP-1 cells (4 × 10^5^ cells/mL) were seeded in 12-well plates and cocultured with 100 μL/well siRNA-lipofectamine mixture in 500 μL Opti-MEM (Gibco, USA) medium for 6 h at 37°C in a humidified atmosphere containing 5% CO_2_. After transfection, cells were cultured in fresh complete RPMI 1640 medium for a further 48 h to allow gene silencing. Target gene silencing efficiency was analyzed by Western blot. The siRNA target gene sequences are listed in Table S2.

10.1128/mbio.02004-22.7 TABLE S2 siRNAs used in this study. Download Table S2, DOC file, 0.03 MB. Copyright © 2022 Mo et al. 2022 Mo et al. https://creativecommons.org/licenses/by/4.0/ This content is distributed under the terms of the Creative Commons Attribution 4.0 International license.

### Western blotting.

Differentiated THP-1 cells (4 × 10^5^ cells/mL) treated with siRNA and/or H37Ra/H37Rv (MOI = 10) were washed with 1× PBS and lysed with lysis buffer (P0013, Beyotime, Shanghai, China). The protein concentration was determined by a bicinchoninic acid (BCA) assay kit (Beyotime, Shanghai, China), and equal amounts of protein from each sample were separated by sodium dodecyl sulfate-polyacrylamide gel electrophoresis (SDS-PAGE, 15% gel). The proteins were then transferred to PVDF membranes (Merck/Millipore) and blocked with PBST (1× PBS with 0.1% Tween 20) containing 5% nonfat dried milk (GBCBIO Technologic Inc. China) for 1 h at room temperature (RT). Blocked membranes were washed twice with PBST and incubated overnight at 4°C with primary antibodies (Table S1). After washing with PBST (≥6 washes, 5 min each with shaking), membranes were incubated with appropriate Horseradish Peroxidase (HRP)-conjugated secondary antibody (Abcam, USA,1:10,000) for 1 h at RT. After washing with PBST, the blots were visualized with SuperSignal West Pico PLUS solutions (Thermo Fisher Scientific Inc., USA) using the MiniChemi imaging system (SAGECREATION, China).

### RNA extraction and quantitative real-time PCR.

Total RNA was extracted from treated THP-1 cells (4 × 10^5^ cells/mL) using RNeasy kits (Omega, USA) according to the manufacturer’s instructions. Contaminating DNA removal and reverse transcription were performed using HiScript II Q RT SuperMix for qPCR (Vazyme, China). Target gene expression was analyzed with the SYBR Green Real-Time PCR Master Mix (Bimake, USA) on the 7500 Fast real-time PCR system (Applied Biosystems, Thermo Fisher Scientific Inc., USA) using the specific primers listed in Table S3 under the following reaction conditions: 95°C 10 min followed by 40 cycles of amplification at 95°C for 15 s and 60°C for 34 s and melting curve analysis at 95°C for 15 s, 60°C for 1 min, 95°C for 30 s and 60°C for 15 s. The relative target gene mRNA expression was normalized to the reference gene (beta-actin or glyceraldehyde-3-phosphate dehydrogenase [GAPDH]) and calculated using the 2^–ΔΔCt^ method.

10.1128/mbio.02004-22.8 TABLE S3 Primers used in this study. Download Table S3, DOC file, 0.03 MB. Copyright © 2022 Mo et al. 2022 Mo et al. https://creativecommons.org/licenses/by/4.0/ This content is distributed under the terms of the Creative Commons Attribution 4.0 International license.

### CFU analysis of infected cells.

Differentiated THP-1 cells (4 × 10^5^ cells/mL) were infected with H37Ra/H37Rv (MOI = 10) for 6 h, washed three times with 1× PBS (Solarbio, China) to remove extracellular bacteria, and then incubated in fresh RPMI 1640 medium for a further 72 h at 37°C under 5% CO_2_. For CFU counting, cells were washed three times with 1× PBS and lysed in 1× PBS containing 0.1% SDS for 10 min at RT. The cell lysate was serially diluted with 1× PBS and 50 μL of each diluted sample was plated on 7H10 agar plates and incubated at 37°C for 15 to 20 days.

### Flow cytometry.

Differentiated THP-1 cells (4 × 10^5^ cells/mL) treated with siRNA and/or H37Ra/H37Rv (MOI = 10) were washed twice with 1× PBS, fixed with 4% formaldehyde (BD Biosciences, USA) at 4°C for 20 min, and then treated with cell membrane breaker (BD Biosciences, USA) at 4°C for 15 min. Cells were then incubated with an anti-HRH1 antibody (R&D Systems, Inc. USA) at 4°C for 50 min. HRH1 expression was determined by flow cytometric measurement of the mean fluorescence intensity (MFI) using a FACSAria II flow cytometer (BD Biosciences, USA), and data were analyzed with FlowJo software version 10 (BD Biosciences, USA).

### Immunofluorescence confocal microscopy.

Differentiated THP-1 cells (2 × 10^5^ cells/mL) treated with siRNA and/or fluorescent GFP-H37Ra/RFP-H37Rv (MOI = 10) for 24 h were then washed twice with 1× PBS, fixed with 4% formaldehyde for 15 min, and permeabilized with 0.3% Triton X-100 for 10 min. After blocking with 3% BSA in 1× PBS for 2 h at RT, the cells were incubated overnight at 4°C with anti-HRH1 (Immunoway, 1:100) and anti-LAMP-1 (lysosomal associated membrane protein 1, Abcam, ab25630, 1:200) antibodies followed by incubation for 1 h at RT with a fluorophore-conjugated secondary antibody (anti-rabbit-Alexa fluor 488/555, Invitrogen, USA,1:200). Before confocal microscopy, cells were Hoechst stained (Invitrogen, USA) and treated with an anti-fluorescence quenching agent (Invitrogen, USA). Confocal images were obtained using an Olympus FV1000 confocal microscope (Nikon A1R) and processed with Image J software (NIH, USA). For the image, 80 to 100 cells in each group were analyzed.

### Immunoprecipitation.

Differentiated THP-1 cells (1 × 10^7^ cells/mL) infected with H37Rv (MOI = 10) for 24 h. Cells were harvested after treatment in NETN buffer containing 20 mM Tris-HCl (pH 7.5), 150 mM NaCl, 1 mM EDTA, 0.5% Nonidet P-40, and protease inhibitor mixture. Anti-HRH1 (ProteinTech, 1:200) and anti-GRK2 (ProteinTech, 1:200) antibodies and IgG were added to cell lysates and incubated overnight at 4°C. After SDS-PAGE, immunoblots were transferred to polyvinylidene fluoride (PVDF) membranes and blocked with PBST (1× PBS with 0.1% Tween 20) containing 5% nonfat dried milk (GBCBIO Technologic Inc. China) for 1 h at RT. Blocked membranes were washed twice with PBST and incubated overnight at 4°C with primary antibodies (Table S1).

### Phagosome acidification analysis.

Differentiated THP-1 macrophages (4 × 10^5^ cells/mL) were treated as described above for 24 h and washed three times with 1× Hanks’ balanced salt solution (HBSS). After adding the pHrodo Red probe (1000×, Invitrogen, USA)/RatioWorks PDMPO probe (1000×, AAT Bioquest, USA) to the HBSS buffer, the cells were incubated for 30 min at 37°C under 5% CO_2_ in the dark and cellular lysosomal acidification was analyzed according to the manufacturer’s instructions. Cellular lysosomal acidification was quantified by flow cytometric measurement of the MFI using a FACSAria II flow cytometer.

### Apoptosis.

Differentiated THP-1 macrophages (4 × 10^5^ cells/mL) were treated as described above for 24 h, washed three times with 1× PBS, and collected by centrifugation at 1,000 × *g* for 5 min at RT. The cells were then resuspended in 195 μL Annexin V-FITC and 5 μL Annexin V-FITC and incubated on ice for 10 to 20 min in the dark. After staining with 10 μL propidium iodide, cell apoptosis was analyzed by flow cytometry using a FACSAria II flow cytometer (BD, USA) and data were analyzed with FlowJo software version 10 (BD Biosciences, USA).

### ROS measurement.

Differentiated THP-1 macrophages (4 × 10^5^ cells/mL) were treated as described above for 24 h and washed three times in a prewarmed serum-free RPMI 1640 medium. Endogenous cytoplasmic ROS (cROS) and mitochondrial ROS (mROS) levels were determined by incubating the cells with 10 μM 2,7-diacetate dichlorofluorescein (H2DCF-DA) and 10 μM MitoSOX Red Mitochondrial Superoxide Indicator, respectively, for 20 min at 37°C in the dark. After incubation, ROS levels were determined by flow cytometric measurement of the MFI using a FACSAria II flow cytometer, and data were analyzed with FlowJo software version 10.

### NADPH oxidase activity measurement.

Differentiated THP-1 macrophages (4 × 10^5^ cells/mL) were treated as described above for 24 h and washed three times in a prewarmed serum-free RPMI 1640 medium. The protein concentration was determined by the BCA assay kit (Beyotime, Shanghai, China). Furthermore, cells were lysed by NADP+/NADPH extract solution, heated at 60°C for 30 min to decompose NADP^+^, and incubated with G6PDH working solution at 37°C for 10 min in the dark. NADPH oxidase activity was quantified using a chromogenic solution and measurement of the absorbance at 450 nm using the EpochTM 2 microplate spectrophotometer (Bio-Tek, USA) according to the manufacturer’s instructions (Beyotime, Shanghai, China).

### Statistical analysis.

All statistical analyses were performed with GraphPad Prism 8.0 (GraphPad Software Inc.). Unpaired two-tailed Student's *t* test was used to analyze the difference between two groups and one-way analysis of variance (ANOVA) was used to compare differences among multiple groups. Data are expressed as the mean ± standard deviation (SD). The threshold for statistical significance was set to *P < *0.05.
