# Supplementary figures and images for: Mycobacterium tuberculosis Utilizes Host Histamine Receptor H1 to Modulate Reactive Oxygen Species Production and Phagosome Maturation via the p38MAPK-NOX2 Axis
Source: mBio. 2022 Aug 24;13(5):e02004-22. doi: 10.1128/mbio.02004-22 (PMC9600773; doi:10.1128/mbio.02004-22)

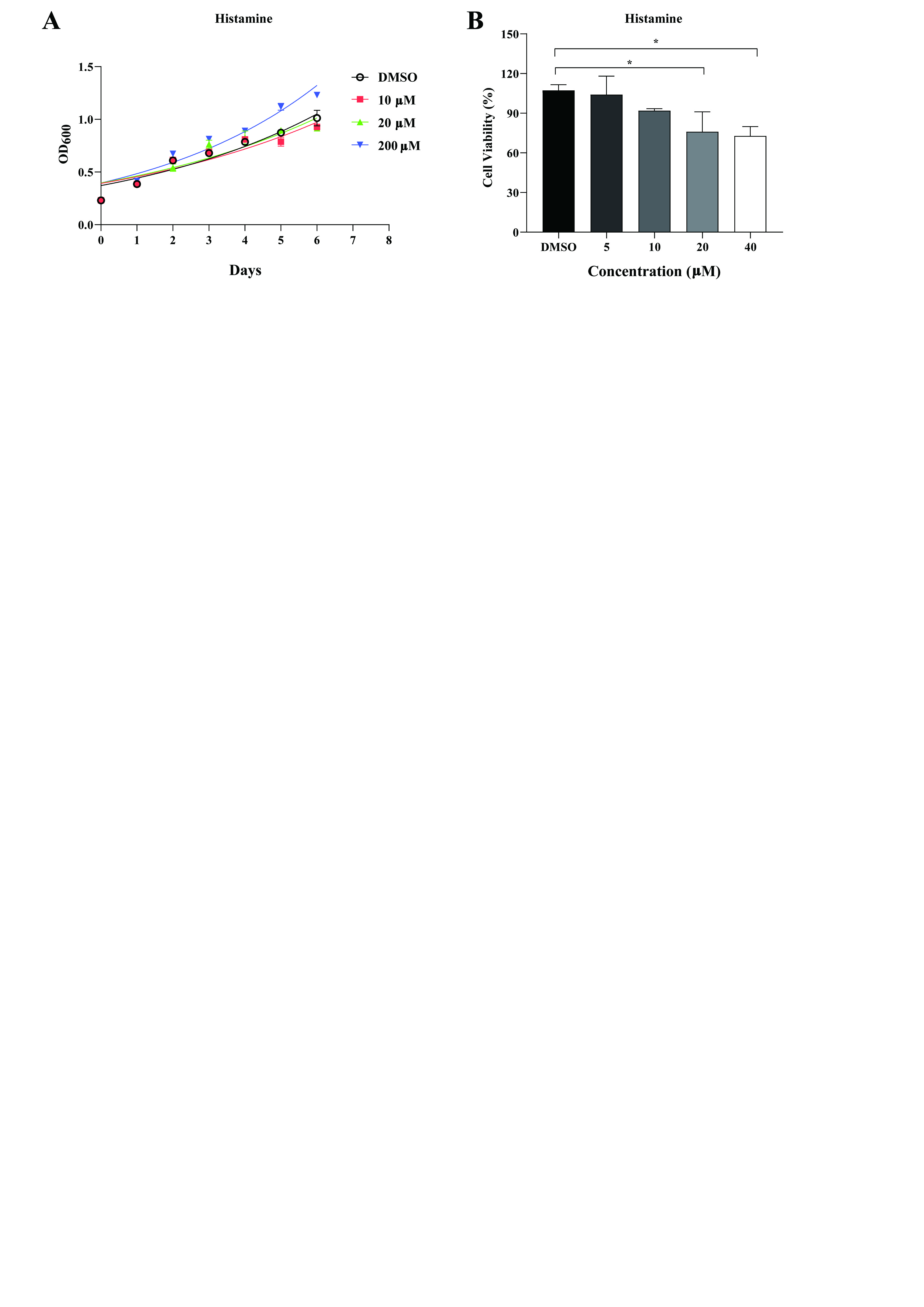

Supplement: FIG S1 [file mbio.02004-22-s0002.jpg]

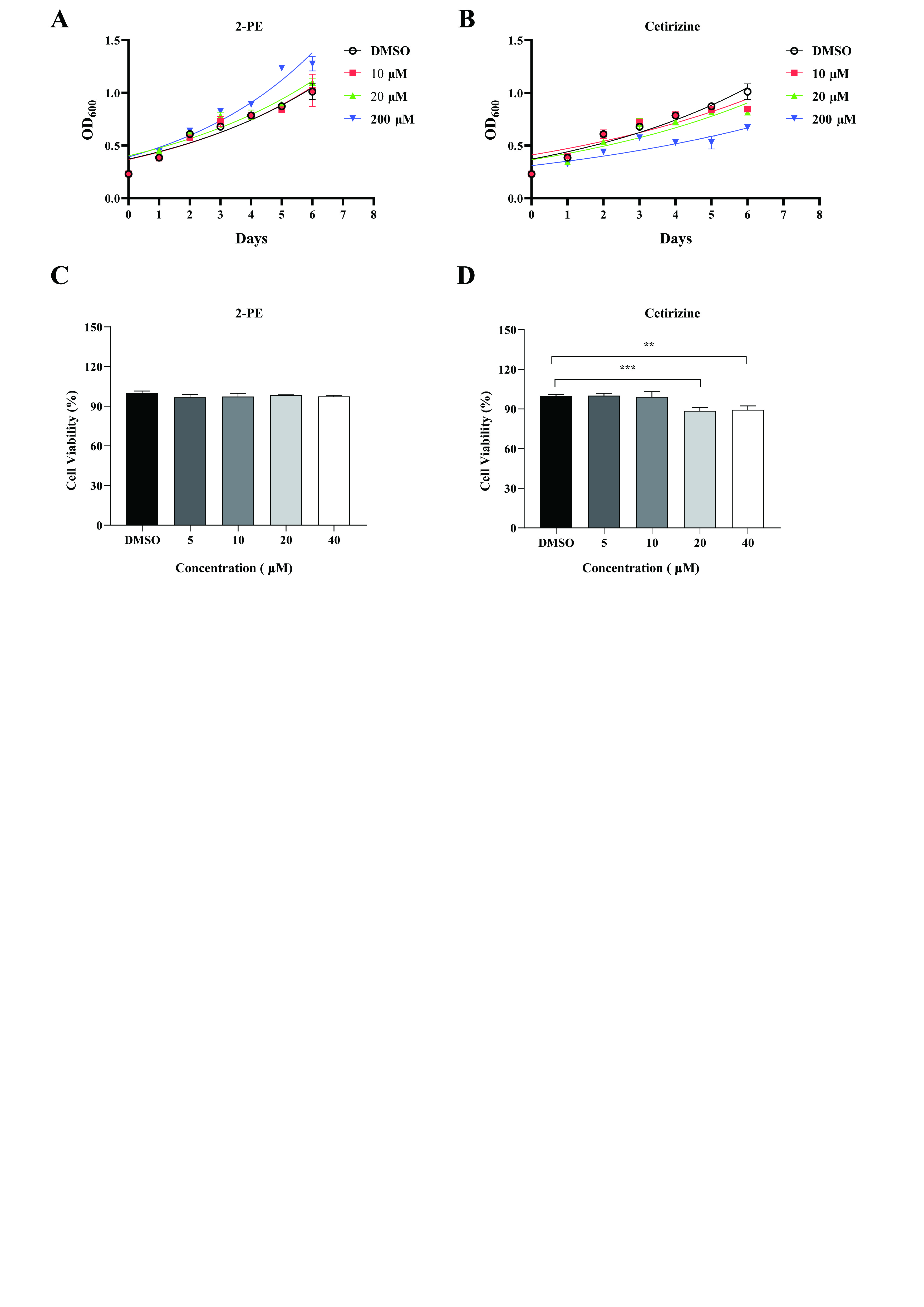

Supplement: FIG S2 [file mbio.02004-22-s0003.jpg]

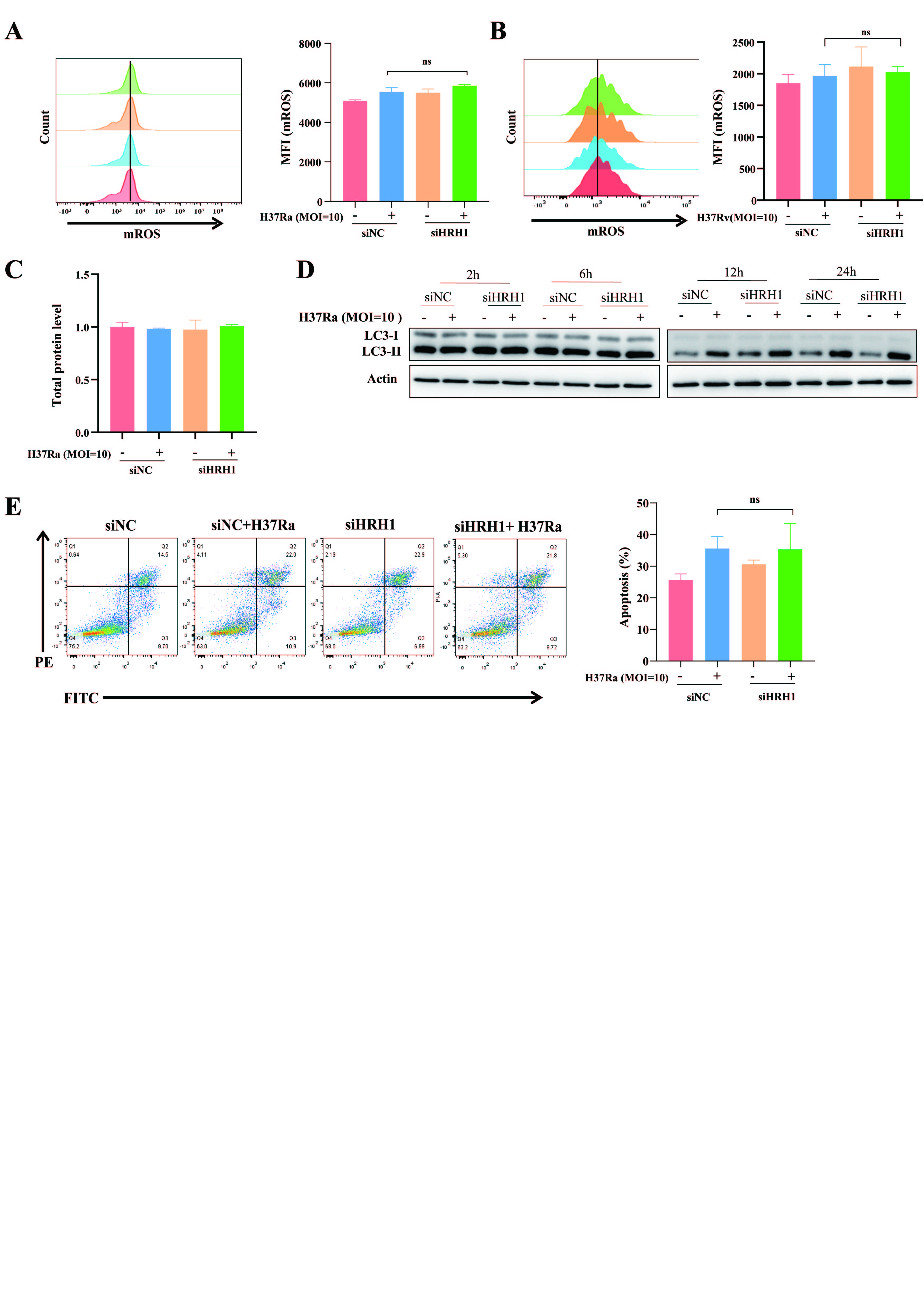

Supplement: FIG S3 [file mbio.02004-22-s0005.jpg]

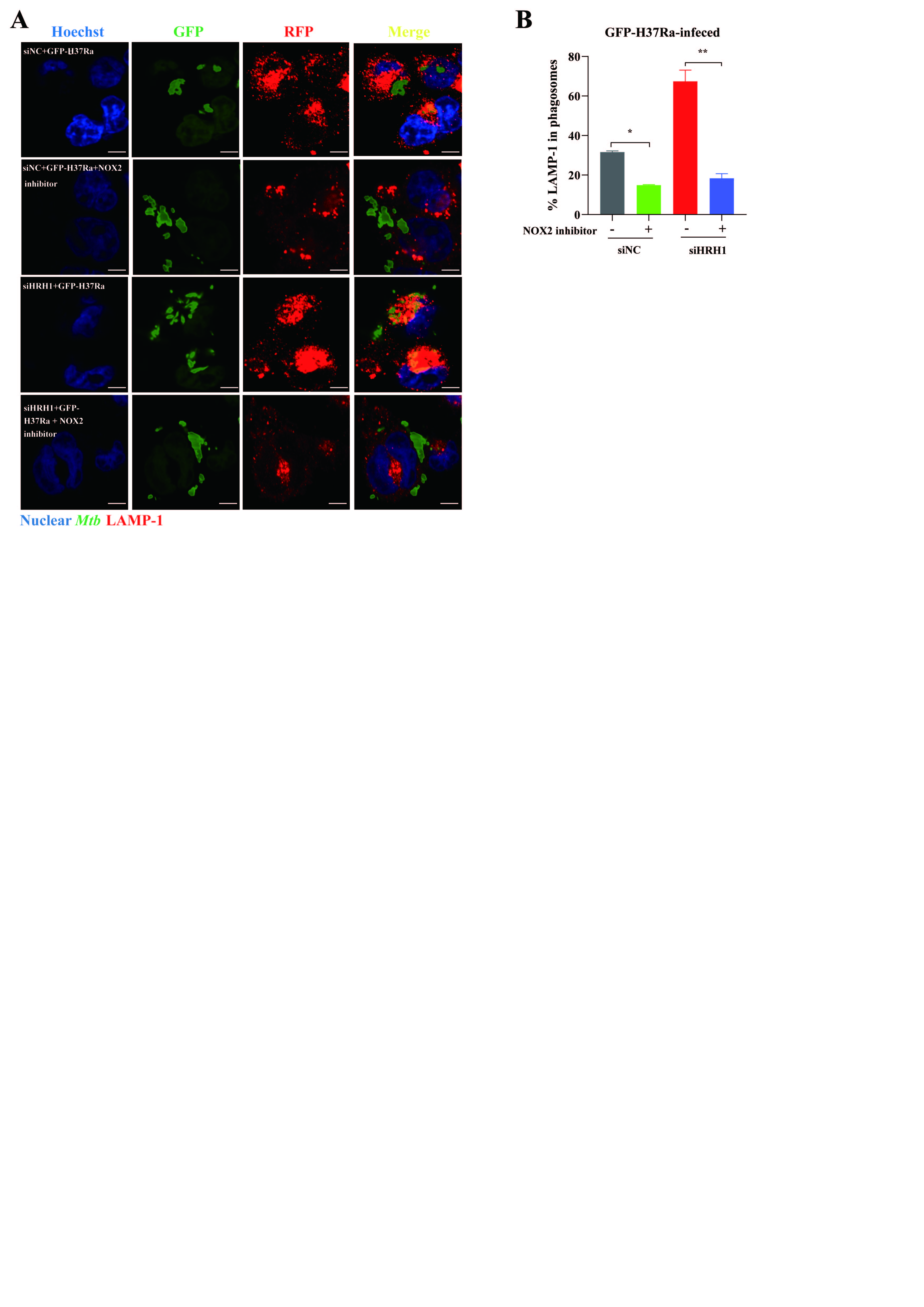

Supplement: FIG S4 [file mbio.02004-22-s0006.jpg]

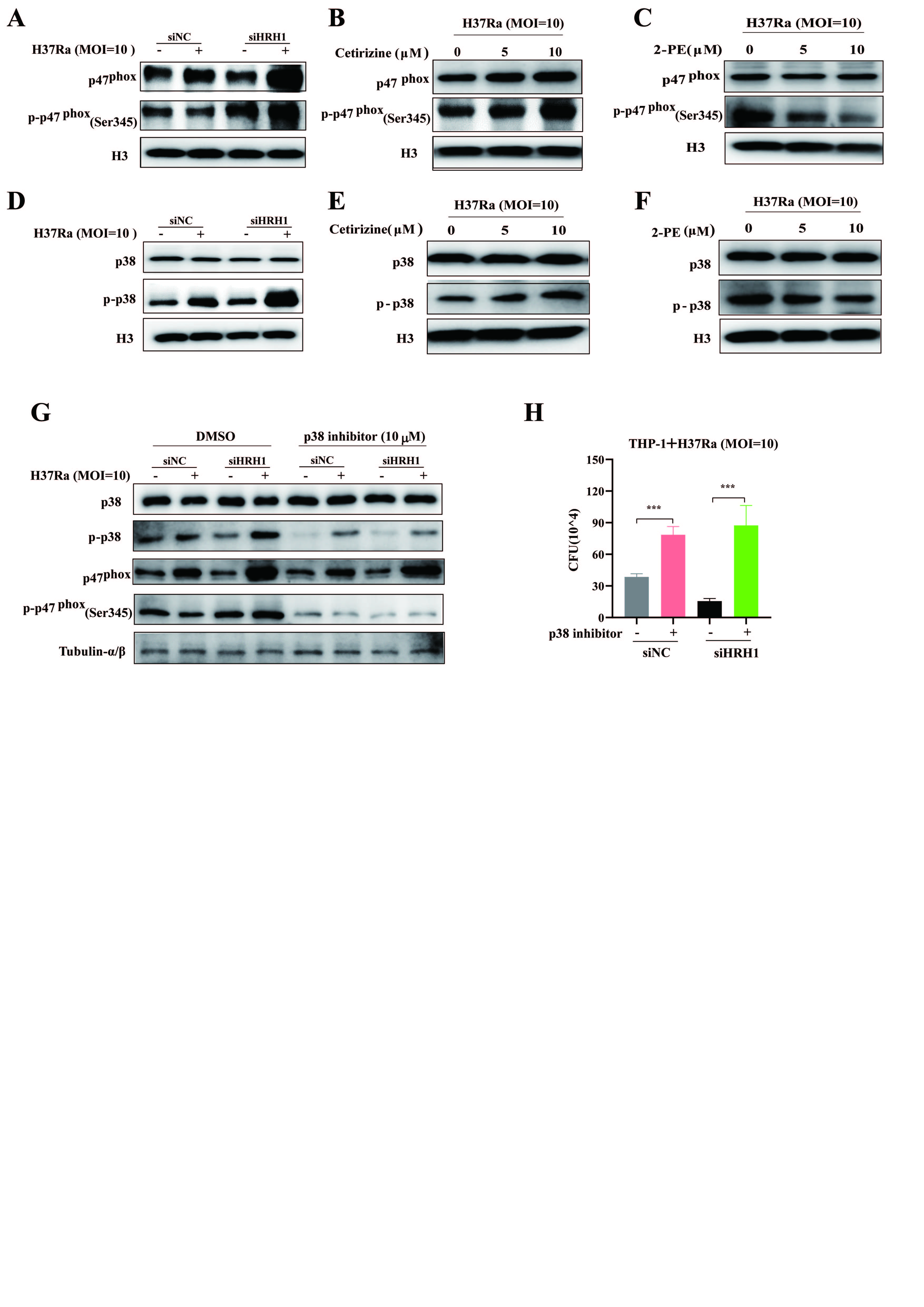

Supplement: FIG S5 [file mbio.02004-22-s0007.jpg]
