# Supplementary material for: Mycobacterium tuberculosis Utilizes Host Histamine Receptor H1 to Modulate Reactive Oxygen Species Production and Phagosome Maturation via the p38MAPK-NOX2 Axis
Source: mBio. 2022 Aug 24;13(5):e02004-22. doi: 10.1128/mbio.02004-22 (PMC9600773; doi:10.1128/mbio.02004-22)
Supplement: TABLE S1 [file mbio.02004-22-s0004.doc]

**Table S1. Antibodies used in** this study.

| Name | Company | Catalog number | Dilution |
| --- | --- | --- | --- |
| HRH1 | Immunoway | YT2140 | 1:1,000 |
| HRH1 | ProteinTech | 13413-1-ap | 1:1,000 |
| HRH2 | Abclonal | A14170 | 1:1,000 |
| HRH3 | Abclonal | A3500 | 1:1,000 |
| HRH4 | Abclonal | A10151 | 1:1,000 |
| GRK2 | ProteinTech | 13990-1-AP | 1:1000 |
| NOX1 | Abclonal | A12309 | 1:1,000 |
| NOX2(gp91^phox^) | ProteinTech | 19013-1-AP | 1:5,000 |
| NOX3 | Abclonal | A3677 | 1:1,000 |
| NOX4 | Abclonal | A3656 | 1:1,000 |
| NOX5 | Abclonal | A7136 | 1:1,000 |
| NCF1(p47^phox^) | GTX (GeneTex) | GTX102957 | 1:1,000 |
| p-NCF1(p-p47^phox^) | GTX (GeneTex) | GTX55367 | 1:1,000 |
| p38MAPK | CST | D13E1 | 1:1,000 |
| p-p38MAPK | CST | D3F9 | 1:1,000 |
| LC3B | CST | 4108S | 1:2,000 |
| Beta-Actin | Abcam | ab179467 | 1:10,000 |
| Tubulin-α/β | CST | 2148S | 1:5,000 |
| H3-histone | Abcam | ab176842 | 1:10,000 |
| Normal Rabbit IgG | Sigma-Aldrich | NI01-100UGCN | 1:1,000 |
