# Supplementary material for: Mycobacterium tuberculosis Utilizes Host Histamine Receptor H1 to Modulate Reactive Oxygen Species Production and Phagosome Maturation via the p38MAPK-NOX2 Axis
Source: mBio. 2022 Aug 24;13(5):e02004-22. doi: 10.1128/mbio.02004-22 (PMC9600773; doi:10.1128/mbio.02004-22)
Supplement: TABLE S2 [file mbio.02004-22-s0008.doc]

**Table S2. siRNA used in** this study.

| Gene name | Number | Target sequence |
| --- | --- | --- |
| *HRH1* | stB0000134C | CTACAAGGCCGTACGACAA |
| *HRH2* | stB0000135B | ATCGTGTCCTTGGCTATCA |
| *HRH3* | stB0000136A | GCGTCACCTTCTTTAACCT |
| *HRH4* | stB0000543A | ATGCCAGATACTAATAGCA |
| *GRK2* | stB0004546A | GGGAGATCTTCGACTCATA |
