# Supplementary material for: Mycobacterium tuberculosis Utilizes Host Histamine Receptor H1 to Modulate Reactive Oxygen Species Production and Phagosome Maturation via the p38MAPK-NOX2 Axis
Source: mBio. 2022 Aug 24;13(5):e02004-22. doi: 10.1128/mbio.02004-22 (PMC9600773; doi:10.1128/mbio.02004-22)
Supplement: TABLE S3 [file mbio.02004-22-s0001.doc]

**Table S3. Primers used in** this study.

| Gene name | Sense (5′–3′) | Anti-sense (5′–3′) |
| --- | --- | --- |
| *HRH1* | CCTGTGCATTGATCGCTACC | GAGGTCTGCTGCATGAAGTG |
| *HRH2* | CGTGTCCTTGGCTATCACTGA | GGCTGGTGTAGATATTGCAGAAG |
| *HRH3* | CCCATACACGCTGCTGATGAT | GGAGGTTTCGTACCAGTAGTCA |
| *HRH4* | ATGCTAGGAAATGCTTTGGTCA | AGGAATGGAGATCACACCCAC |
| *Beta-ACTIN* | CACGATGGAGGGGCCGGACTCATC | TAAAGACCTCTATGCCAACACAGT |
